# Recent Advances in Nanoparticle-Based Optical Sensors for Detection of Pesticide Residues in Soil

**DOI:** 10.3390/bios13040415

**Published:** 2023-03-23

**Authors:** Chunhong Zhang, Mingle Qiu, Jinglin Wang, Yongchun Liu

**Affiliations:** 1 Xi’an Key Laboratory of Advanced Control and Intelligent Process, School of Automation, Xi’an University of Posts & Telecommunications, Xi’an 710121, China; 2 Key Laboratory of Applied Surface and Colloid Chemistry, Ministry of Education, School of Chemistry and Chemical Engineering, Shaanxi Normal University, Xi’an 710062, China

**Keywords:** nanoparticle, sensor, soil, pesticide residues, LSPR

## Abstract

The excessive and unreasonable use of pesticides has adversely affected the environment and human health. The soil, one of the most critical natural resources supporting human survival and development, accumulates large amounts of pesticide residues. Compared to traditional spectrophotometry analytical methods, nanoparticle-based sensors stand out for their simplicity of operation as well as their high sensitivity and low detection limits. In this review, we focus primarily on the functions that various nanoparticles have and how they can be used to detect various pesticide residues in soil. A detailed discussion was conducted on the properties of nanoparticles, including their color changeability, Raman enhancement, fluorescence enhancement and quenching, and catalysis. We have also systematically reviewed the methodology for detecting insecticides, herbicides, and fungicides in soil by using nanoparticles.

## 1. Introduction

To ensure global food security, pesticides have been widely used in agriculture since the second half of the 19th century to improve food production and quality. In recent decades, agricultural producers have often used a large number of highly toxic pesticides that can leave residue to control crop diseases, insects, grasses, and plant growth in order to pursue economic interests. Approximately 2.66 million tons of pesticides were applied worldwide in 2020, according to statistics [1]. Compared to 2000, the use of pesticides in agriculture is predicted to double by 2050, according to Oberemok et al. (2015) [2]. As pesticides are used extensively and unreasonably, their impacts on the environment, food chain, biodiversity, pollination media, and human health are becoming more apparent. Pesticides have been reported to reduce soil respiration by 35% [3]. It is estimated that 90% of farmland water sources are contaminated with pesticides, which threatens aquatic and terrestrial food chains and destroys biodiversity. Even at environmentally safe doses, pesticide exposure reduces species richness by 42% in Europe [4]. Furthermore, pesticide residues threaten the survival of animal pollinators such as bees and butterflies. Over half of the native bee species are threatened in North American regions because of excessive pesticide use [5]. The most important thing is that pesticide residues negatively affect human health. Pesticide residues can cause serious diseases such as cancer, endocrine disruption, neurological damage, reproductive dysfunction, and other health problems [6]. For these reasons, pesticide residue studies are a practical and theoretical necessity for reducing the use of pesticides and protecting human health.

The soil is not only a vital resource for human survival and development but also a necessary living environment for most plants. Because soil is the final destination for pesticides, a great deal of pesticide residue has accumulated in it. In the process of spraying pesticides, only about 10% of the pesticides are left on the crops [7]. Most of the pesticides are sprayed into the soil or washed into the soil by rain, and pesticides left on the crops may also fall into the soil with branches and leaves [8]. Additionally, some pesticides degrade slowly in soil and bind strongly to it, such as pyrethroids [9]. In addition to absorbing and enriching pesticides, soil also reduces its degradation rate due to clay minerals, colloidal particles, and organic matter [10]. As a result of these reasons, a large amount of pesticide accumulates in the soil, making soil pesticide pollution urgent to monitor [11].

In comparison with other samples, soil residues of pesticides display these characteristics: (1) Serious harm. Pesticide residues can greatly disrupt ecosystems by destroying microbial populations, bacterial diversity, nitrogen transformation, enzymes, and animals in the soil. Furthermore, they will threaten our health directly or indirectly, such as via skin contact or dust inhalation, or even through groundwater pollution or agricultural irrigation into rivers, putting our drinking water at risk [12]. (2) There are numerous varieties. Almost all pesticides will ultimately enter the soil. In addition to pesticides applied directly to the soil, pesticides sprayed on the leaves of plants will also eventually enter the soil during the spraying process or as rain washes off or leaves fall, resulting in many kinds of pesticide residues in the soil. (3) Complex components. The soil is composed of many organic and inorganic components, such as humus (complex macromolecules formed by the oxidation and polymerization of organic compounds), clay minerals, and colloidal particles. Due to these characteristics, soil pesticide residue detection is not only significant but also places higher demands on detection methods’ sensitivity, specificity, and anti-interference abilities.

The traditional methods for detecting pesticides in soil include high-pressure liquid chromatography, spectrophotometry with solid-phase extraction, and ion mobility spectrometry with headspace solid-phase microextraction [13]. In spite of their high accuracy, these methods have some shortcomings, including unsatisfactory detection limits; complex, time-consuming analyses; the requirement of highly trained operators; and high costs. For many years, researchers have been devoting themselves to developing sensitive, cheap, and user-friendly sensing systems for pesticides in soil. In addition to other sensing strategies, nanoparticle-based detections are desirable because of their unique optical or catalytic properties. In recent years, researchers have been working on developing inexpensive, sensitive, and user-friendly soil pesticide detection systems. Nanoparticles have unique optical and catalytic properties, making them an attractive option among various sensing strategies. Furthermore, as nanoparticles are simple to combine with other recognition molecules, such as antibodies, aptamers, MIP, etc., they provide high specificity to pesticides, which eliminates the need to extract soil samples for pesticide analysis. Pesticide residues are detected using a variety of nanoparticles, including gold, silver, iron oxide, zirconia, titanium dioxide, silicon dioxide, carbon nanotubes, graphene nanoparticles, QDs, and so on [14]. Based on this, colorimetry, SERS, fluorescence, electrochemistry, and chemiluminescence were proposed as detection methods with high sensitivity and ease of operation. Over the past 20 years, these methods have been extensively studied, and important progress has been made.

**Figure 1:**
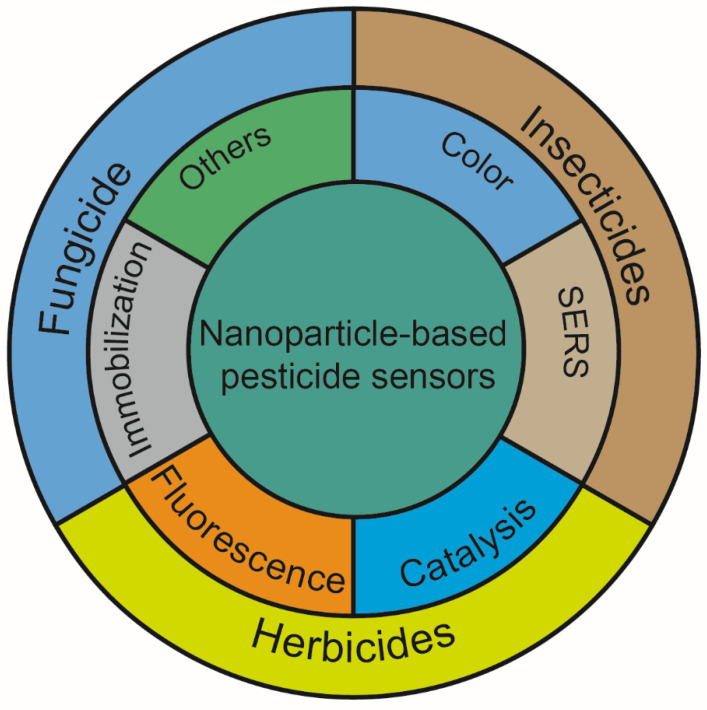
The functions of nanoparticles and their application in soil pesticide detection.

In this review, we focus on methods for detecting pesticide residues in soil using nanoparticles. There is an overview of the detection principles and how they are applied in the detection of various pesticides. In particular, the functions of nanoparticles in pesticide detection were discussed, such as changeable colors, SERS, fluorescence enhancement or quenching properties, catalysis, and so on. Moreover, their application to the detection of insecticides, herbicides, and fungicides was also summarized (Figure 1).

## 2. The Role of Nanoparticles in the Detection of Pesticides

### 2.1. Unique and Variable Color

As light interacts with a noble metal nanoparticle, the oscillating electric field couples with the electrons on the surface, causing them to collectively oscillate at the same frequency. This phenomenon is known as localized surface plasmon resonance (LSPR). At the resonant wavelength, LSPR can lead to a strong electromagnetic field near the surface of the nanoparticle and thereby cause extinction. The characteristics of LSPR are affected by the morphology, size, aggregates, and surrounding medium of the nanoparticles. Additionally, the electromagnetic field is maximal at the particle surface but decreases rapidly with distance from the surface. The LSPR-induced local electric field is a crucial factor in surface-enhanced Raman scattering [15] and surface-enhanced fluorescence [16].

#### 2.1.1. The Original Color of the Nanoparticles

**Figure 2:**
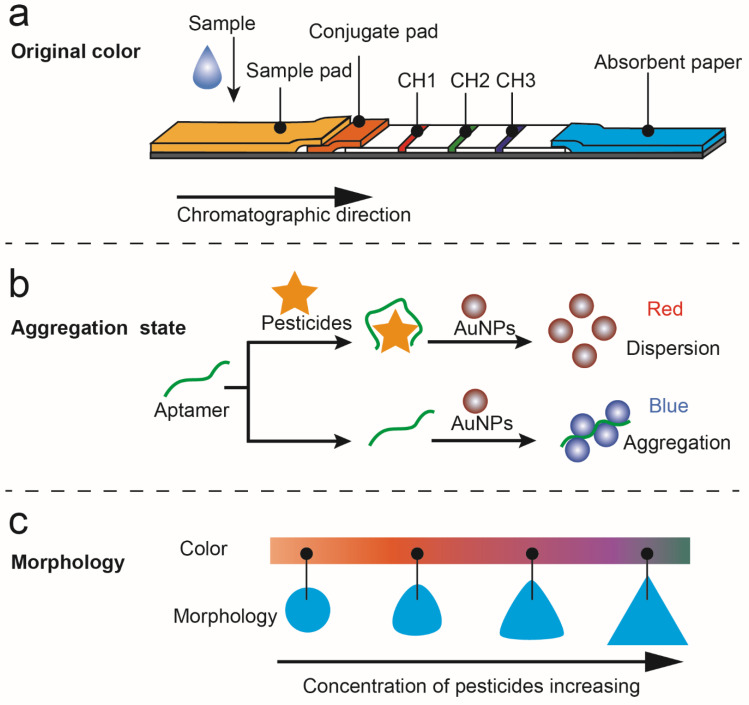
Detection of pesticides based on the unique and variable color of nanoparticles. (**a**) Illustration of the lateral flow immunoassay based on the use of gold nanoparticles for simultaneous detection of three pesticides [17]. (**b**) A schematic illustration of the aptamer-based colorimetric sensing of acetamiprid [21]. (**c**) The mechanism of anti-etching colorimetric detection based on TSNPs for thiram residues in soil [23].

The LSPR effect of noble metal nanoparticles produces a specific color associated with the extinction peak, which can be used to determine the concentration of analytes. The intrinsic color of nanoparticles was always used in the test strip method to indicate pesticide content. Using colloidal gold and three competitive immunoreactions, an immunoassay that simultaneously detects imidacloprid, chlorpyrifos-methyl, and isocarbophos was developed by Wang et al. [17]. The monoclonal antibodies were conjugated with colloidal gold. On the conjugate pad, three monoclonal antibody-modified gold conjugations were dispensed. A competitive immunoreaction took place in each channel as the sample solution flowed through the test strip. The analytes in the sample will bind first to the antigen-modified gold conjugates, so that the colloidal gold cannot be captured by the coating antigens and thus appears colorless. Using four strips of lateral flow immunoassay with different concentrations of capture reagent, semiquantitative results can be obtained (Figure 2a).

#### 2.1.2. The Color Change Caused by the Aggregation of Nanoparticles

The colloid stability of nanoparticles is closely related to the Waals attraction energy between particles and charge on particles, which can be destroyed by coordination bond interaction, adsorption, or salt ions, resulting in color changes caused by the aggregated nanoparticles.

A novel methyl parathion colorimetric sensor was developed based on the coordination bond interaction between lanthanum-functionalized AuNPs and methyl parathion. When methyl parathion is introduced into the system, insoluble lanthanum phosphate is produced, causing AuNPs to aggregate and change their color from red to blue [18]. Additionally, the surface atoms on the Ag and Au nanoparticles and the sulfur atom in pesticides can also serve as ligands that bind via Ag/Au–S bonds. Consequently, the surface stabilizer (e.g., citrate groups) surrounding the nanoparticles was displaced, resulting in aggregation and color changes [19].

“Artificial antibody” aptamers exhibit high affinity, specificity, and selectivity for their target molecules, so they have been widely applied in sensors for recognizing various targets. Using AuNPs and an acetamiprid-binding aptamer as recognition elements, Cao et al. realized acetamiprid detection selectively and sensitively. The combination of the target and the aptamer can promote the salt-induced aggregation of AuNPs, and thus the specific color changes caused by the interparticle plasmon coupling can be obtained [20]. However, the salt-induced process may have the disadvantage of increasing experimentation steps and affecting aptamer-pesticide affinity. Hou et al. reported an aptamer-based colorimetric analysis of AuNPs without introducing salt (Figure 2b). Positively charged AuNPs can respond directly and sensitively to aptamer conformational changes induced by acetamiprid, leading to the aggregation of AuNPs and changing their appearance color. This method requires no salt and avoids the problem of using negatively charged AuNPs in colorimetry [21].

In real samples, however, nanoparticle aggregation is usually influenced by other factors, such as surfactant, pH, ion concentration, etc. In order to overcome this challenge, a method of anti-aggregation of nanoparticles for colorimetric detection has been proposed. For example, thiram and Ag^+^ can compete with each other in triggering the aggregation of gold nanoparticles encoded by 4-aminothiopheno. A stable complex is formed between thiram and Ag^+^, thereby allowing the modified nanoparticles to be well dispersed. By measuring the color change of the nanoparticle probe, thiram can be detected [22].

#### 2.1.3. The Color Caused by Morphology Change of Nanoparticles

The changes in nanoparticle morphology that result from etching have a significant effect on their LSPR, which can be applied to pesticide sensing. We previously found that the morphology of TSNPs can be etched by I^−^; at the same time, thiram can bind to TSNPs by an Ag–S bond to protect them against the etching of I^−^. By regulating the antagonistic interaction between thiram’s protection and I^−^’s etching, a TSNP-based anti-etching colorimetric detection method was developed that is resistant to interference from other substances, including other pesticides and halides (Figure 2c) [23].

### 2.2. SERS

Raman spectroscopy can analyze the vibration and rotation of molecules to determine their structure and concentration. Using noble metallic nanoparticles in SERS to detect pesticides in soil has become increasingly popular in recent years with the development of nanotechnology. As a result of the LSPR properties of these nanoparticles, they can cause a strong local electric field enhancement, which can enhance Raman signals and reduce the LODs of SERS [24].

**Figure 3:**
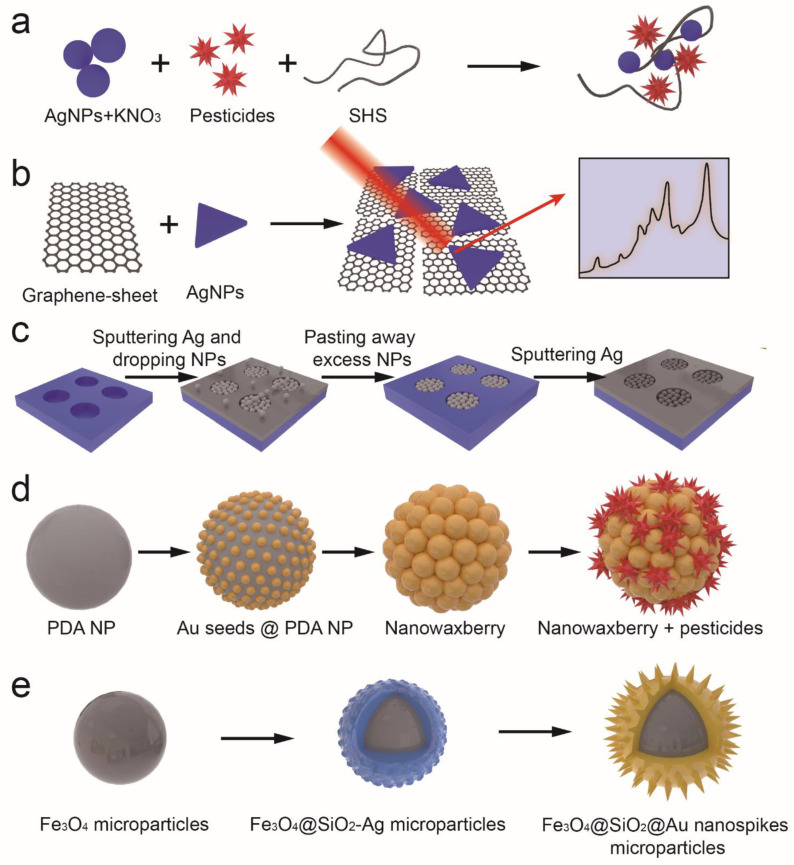
Detection of pesticides based on the SERS of nanoparticles (**a**) Soil humic substances-assisted AgNPs SERS substrates for the detection of s-triazine pesticides [27]. (**b**) Preparation of Ag-nanoplate decorated graphene-sheet SERS substrates for the detection of organic pesticides [29]. (**c**) Magnetic assembled 3D SERS substrate for sensitive detection of pesticide residue in soil [30]. (**d**) Polydopamine gold nanowaxberry-based SERS substrate for detection of pesticides [32]. (**e**) Dense Au nanospikes grown on magnetic microparticles for the detection of pesticides [34].

Gold and silver nanoparticles are widely used as SERS substrates since they are simple to prepare. Nie et al. synthesized AuNPs of different sizes as SERS substrates to determine chlorpyrifos residues in soil. According to the results, chlorpyrifos SERS characteristic peak intensity and AuNPs size are linearly related [25]. They also used AuNPs as the SERS substrate to detect deltamethrin and carbofuran in soil [26]. With regard to AgNPs, Sanchez-Cortes et al. found that prometryn and atrazine in the soil can be detected by using AgNPs as SERS substrates (Figure 3a) [27]. Additionally, this method can be used to analyze DEHA quantitatively [28]. Since surface charges tend to accumulate at the tips of nanoparticles, nanostructures with sharp tips enhance electric fields more than those with spherical nanoparticles. In our previous work, TSNPs with small sizes and sharp corners were used as SERS substrates to detect thiram residue in the soil. Interestingly, the smaller TSNPs perform better in SERS [13].

An increase in the density of self-assembly particles can change the arrangement and distance between nanoparticles, therefore improving the SERS performance. Lei et al. constructed a graphene/Ag-nanoplate hybrid SERS substrate that allows Ag-nanoplates to create “hot spots” for the amplification of SERS signals. In addition, the strength of the interaction between the graphene sheet and the pesticides can facilitate the assembly of pesticides. Thus, thiram and methyl parathion can be detected with high sensitivity using this method (Figure 3b) [29]. Zhang et al. created a self-assembling 3D SERS substrate using a magnetic force. As the “hot spots” appeared throughout the 3D SERS substrates, their sensitivity was higher than that of conventional SERS substrates with 2D plasmonic structures (Figure 3c) [30]. A bottom-up SERS substrate based on rough gold nanorods with different particle densities was fabricated by Cong et al. A layer of rough gold nanorods on a Si wafer is bulked out by electrostatic interaction, then built up layer by layer by interface self-assembly. As particle density increases, the SERS intensity increases dramatically, and rough gold nanorods produce a stronger electromagnetic field enhancement than smooth gold nanorods. Using the optimal SERS substrate, soil thiram can be quantified quantitatively [31].

The nanocomposites are also used as SERS substrates in order to achieve more “hot spots”. For example, the polydopamine gold nanowaxberry was prepared via seed-mediated synthesis (Figure 3d). Firstly, the polydopamine sphere is covered with Au seeds. Afterward, an I- ion coordinating ligand is used to form a stable AuI_4_^−^ complex for decreasing AuCl_4_^−^’s reduction potential so that Au nanoparticles are deposited with high density and uniformity on the surface of polydopamine spheres to create nanowaxberries. These nanowaxberries with a high density of holes and gaps in three-dimensional space could adsorb analytes and benefit practical SERS analysis [32]. Liu et al. used Cu_2_O nanooctahedrons and intertwined Ag nanovines to construct a heterostructure for detecting thiram in soil. Ag NVs are positioned between adjacent Cu_2_O octahedrons in order to promote electron and hole separation, reduce the recombination of photogenerated carriers, and improve chemical enhancement. The accumulation of electrons on plasmonic NVs can improve electromagnetic enhancement by optimizing the collective oscillation of electrons. Consequently, the SERS activity of the Ag NVs/Cu_2_O heterostructures obtained is 2.7 and 7.0 times greater than that of monodispersed Ag or Au NPs modified Cu_2_O [33]. As a result of their ability to integrate magnetic manipulation and SERS sensing, magnetic/plasmonic microparticles have attracted considerable attention. In Wang et al.’s study, dense Au nanospikes were grown on magnetic microparticles via seed/ligand cooperative growth. By using Dopa-mediated heterogeneous deposition, one is able to create a smooth gold seed layer on magnetic particles modified by silver nanoparticles. In this way, the Ag ions released by magnetic particles form Dopa-Ag^+^ capped ligands around the gold seed layer, which guide all gold seeds to produce dense gold nanoneedles in a synchronous manner. Because of the rich hot spots and magnetic response of the prepared magnetic gold particles, they exhibit excellent SERS sensitivity and can be used to detect traces of thiram in complex samples (Figure 3e) [34].

Additionally, Chen et al. described a method for detecting thiram residue with simultaneous extraction and fabrication of SERS substrates. SERS substrates are prepared by embedding thiram-trapped AuNPs into cellulose p-toluenesulfonates films through the evaporation of dichloromethane after they have been diffused into it. This film can provide an internal standard SERS signal to calibrate the absolute thiram signal, eliminating the fluctuation of SERS intensities and achieving reliable quantitation [35].

### 2.3. Fluorescence Enhancement or Quenching Characteristics

The surface-enhanced fluorescence effect is the phenomenon in which the fluorescence emission intensity of the fluorescent substance at the surface of noble metal nanoparticles is much stronger than its intensity in the free state [16]. Similar to the SERS effect, the local electric field enhancement caused by the LSPR of noble metal nanoparticles makes the molecules near the surface of the particles activate, which improves the excitation efficiency and enhances the intensity of fluorescence emission. Different from the SERS effect, the SEF effect is cross-space. Because the substrate has the dual effect of quenching and enhancing the fluorescence emission, the fluorescence of the substance can be enhanced only when there is a certain distance between the fluorescent substance and the surface of the particles. When the distance is very close (<5 nm), the dipole interaction between the excited fluorescent molecules and the noble metal nanoparticles can transfer the energy to the noble metal nanoparticles in the form of non-radiation, so that the fluorescence emission of the fluorescent molecules is quenched. When the distance is appropriate (5–20 nm), the fluorescence emission intensity of fluorescent substances will be significantly enhanced. However, when the distance between them is too large (>20 nm), the fluorescence enhancement effect will be weakened [36]. The SEF effect usually has ultra-high sensitivity and is widely used in biological detection and optical sensing.

**Figure 4:**
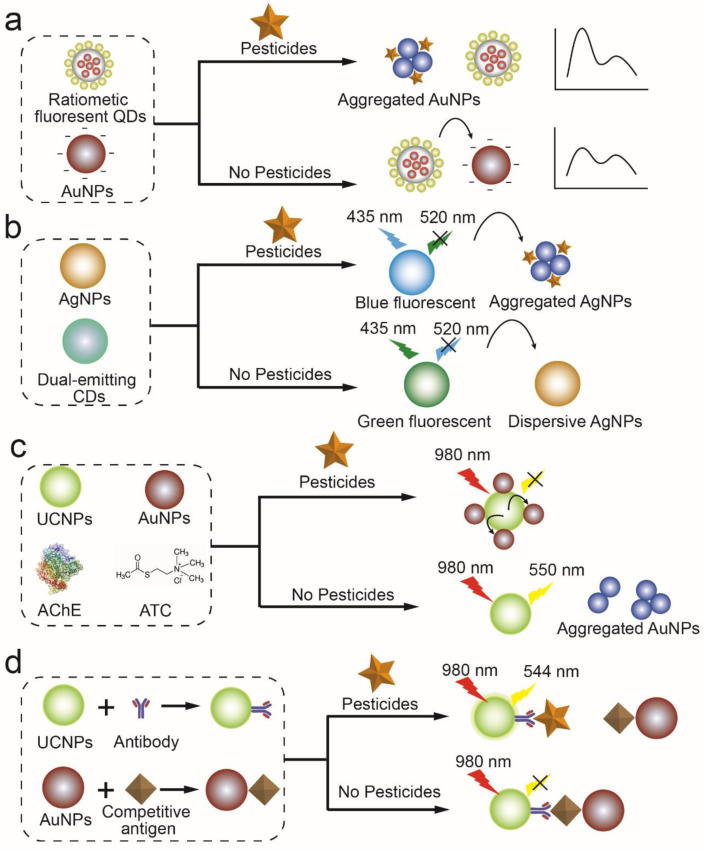
Detection of pesticides based on the fluorescence enhancement or quenching of nanoparticles (**a**) IFE indicated an acetamiprid sensor based on AuNPs on QDs [37]. (**b**) fluorometric dual-channel ratiometric determination of the fungicide cymoxanil based on an analyte-induced aggregation of AgNPs and dual-emitting CDs [38]. (**c**) UCNPs-AuNPs-based FRET assay for the detection of organophosphorus pesticides [36]. (**d**) immunoassay of acetamiprid by using IFE of AuNPs on UCNPs [41].

As a non-radiative energy conversion model, the IFE is easy to achieve. Whenever the absorber’s absorption spectrum overlaps the fluorescer’s emission spectrum, light from the fluorescer can be absorbed. A characteristic of RF-QDs that can be quenched by AuNPs based on IFE enabled Su et al. to detect acetamiprid visually and fluorescently. Adsorption of Acetamiprid on the surface of AuNPs could cause the AuNPs to aggregate, thereby weakening the IFE of AuNPs on RF-QDs and increasing the photoluminescence of RF-QDs (Figure 4a) [37]. Gui et al. found that AgNPs can reduce the blue fluorescence in the dual (blue and green) fluorescence emitted by the CDs through IFE. When cymoxanil is added to the mixture of CDs and AgNPs, the aggregation of AgNPs is triggered by cymoxanil because of electrostatic attraction and hydrogen bonding interactions. A red shift is evident in the absorption spectrum of AgNP aggregates, which overlaps the green emission of CDs, resulting in an IFE on green fluorescence and the recovery of blue fluorescence. Thus, a unique dual-channel ratiometric method is constructed to realize the detection of cymoxanil (Figure 4b) [38].

When the distance between two fluorophores (donors) and nanoparticles (acceptors) is small, FRET occurs between them. In this way, pesticides could be detected by changing the distance between fluorophores and NPs. Based on this principle, Yao et al. developed a nanosensor for OPs based on FRET between UCNPs and AuNPs (Figure 4c). Because ATC hydrolyzes into thiocholine, quenching UCNPs’ fluorescence, OPs are detected based on changes in fluorescence intensity caused by their inhibition of AChE [36]. It is also possible to develop FRET-based sensors by inhibiting FRET with pesticides and fluorophores in competition. For example, RhB could be adsorbed on the surface of Au/Fe_3_O_4_ NPs through electrostatic interaction, resulting in FRET between them. As malathion hydrolysate with -SH forms a stronger Au–S bond than RhB, it competes with RhB for desorption from Au/Fe_3_O_4_ NP surfaces, recovering fluorescence emission [39].

In addition to electrostatic interaction, DNA or antigen-antibody interactions can also affect the distance between the fluorophore and quenchant. Using gold nanoparticles as fluorescence quenchers, Yang et al. developed a DNA-modified nanobeacon for determining OPs. Nanobeacons display efficient quenching of fluorescence due to the presence of fluorophore and quencher (AuNPs) close to each other in the stems of hairpin structures formed by DNA. Hybridizing ssDNA with the Au-nanobeacon results in a conformational reorganization of the Au-nanobeacon. In this way, the fluorophore and gold nanoparticles keep their distance from each other, restoring the fluorescence signal. When OPs are present, ssDNA binds to OPs preferentially, reducing hybridization between ssDNA and the Au-nanobeacon. Fluorescence intensity decreases with OP abundance, making OP determination possible [40]. Wang et al. determined acetamiprid using the interaction between antigen and antibody. Without acetamiprid, AuNP-conjugated antigen can combine with UCNP-conjugated mAb, quenching the fluorescence. The fluorescence recovery was observed after acetamiprid inhibited the combination of antigen-AuNPs and UCNPs through the interaction between antibodies and acetamiprid (Figure 4d) [41].

### 2.4. Catalytic Characteristics

Various inorganic nanoparticles can act as catalysts similar to enzymes, which are called nanozymes. In comparison with natural enzymes, nanozymes have higher environmental stability and lower production, purification, and storage costs [42]. These nanozymes display significant catalytic enhancements in comparison to their bulk counterparts, including enhanced reactivity and selectivity [43]. By controlling the size, shape, composition, and surface properties of nanozymes, their catalytic activity can also be fine-tuned [44]. Additionally, the nanozyme has a larger geometric surface area than its active surface area, which makes it an ideal candidate for surface binding reactions controlled by kinetics.

**Figure 5:**
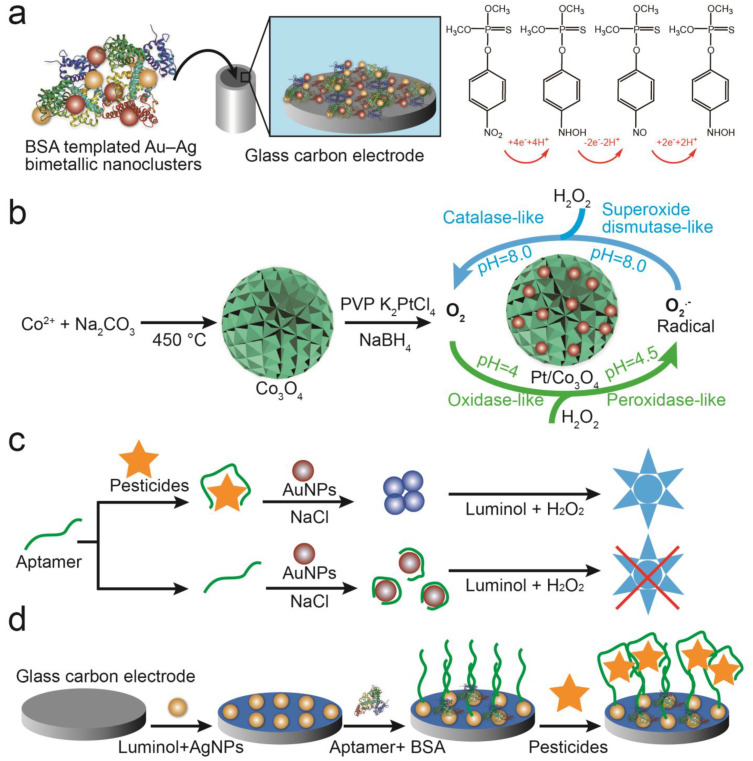
Detection of pesticides using nanoparticle catalytic properties. (**a**) Using AuNPs and MWCNTs-CeO_2_-modified electrodes to catalyze redox reactions of methyl parathion [46]. (**b**) A multifunctional colorimetric detection platform using Pt/Co_3_O_4_ to detect thiram [52]. (**c**) An AuNP-based chemiluminescence sensor to detect acetamiprid based on conformational changes in aptamers [53]. (**d**) AgNPs modified with aptamers for detecting atrazine by ECL [54].

Analyzing the change in the electrochemical signal produced by pesticide redox reactions catalyzed by nanoparticle-modified electrodes provides a reliable method of pesticide detection. Ai et al. [45] and Bagheri et al. [46] modified the electrode with AuNPs, MWCNTs-CeO_2_ composites, and BSA-template Au–Ag bimetallic nanoclusters (Figure 5a), respectively. Modified electrodes efficiently catalyze the redox reactions of methyl parathion, and methyl parathion in the soil can be detected electrochemically. Using PdNPs-modified screen-printed electrodes, Wu et al. demonstrated outstanding electro-catalytic activity for carbendazim determination in soil [47]. Additionally, Bloat and Abaci produced a composite film consisting of AuNPs, ionic liquid, and chitosan for single-use electrodes. As a result of the high conductivity of the ionic liquid, the biocompatibility and film-forming abilities of chitosan, as well as AuNPs’ high electrocatalytic activity and good adsorption capabilities for thio-containing groups, a synergistic effect was achieved. NPs not only increased the electrode’s specific surface area but also enhanced the electrochemical response to malathion without the help of AChE [48].

The inhibition of the catalytic action of nanoparticles by pesticides is also a widely used method for detecting pesticides in soil. For instance, a combination of AuNPs and H_2_O_2_ can oxidize o-phenylenediamine to 2,3-diaminophenazine, which has a yellow color and a typical absorption peak at 450 nm. A relationship is established between dimethoate amounts and solution color since dimethoate can strongly inhibit AuNP catalysis [49]. Sharma et al. utilized Ag_3_PO_4_ nanoparticles’ oxidase-mimicking properties to detect chlorpyrifos fast and selectively. In the presence of Ag_3_PO_4_ NPs nanozymes, chlorpyrifos is oxidized to produce sulfide ions and chlorpyrifos oxons. Through feedback inhibition, sulfide ions in the reaction system can inhibit Ag_3_PO_4_ catalysis for TMB by sensing chlorpyrifos pesticides [50]. In addition to enhancing electron transfer between target analytes and electrode surfaces, CuO NPs can also inhibit catalysis by pesticides [31]. By modifying glassy carbon electrodes with CuO NPs-deposited waste coffee grounds and activated carbon, Mukdasai et al. developed a simple and novel enzyme-free electrochemical sensor for the detection of methyl parathion. CuO NPs on the electrode readily bind methyl parathion and inhibit redox reactions, thereby decreasing Cu’s redox peak current and making the electrode an excellent electrochemical catalyst for the determination of methyl parathion [51].

Analyzing the signals of substrates catalyzed by nanoparticles can also be used to detect pesticides in soil. Rao et al. [52] established a multifunctional colorimetric detection platform for thiram and Cu^2+^ by utilizing the excellent oxidase activity of Pt/Co_3_O_4_. By oxidizing TMB to oxTMB, the colorless reaction system changed to blue. After the addition of thiram, the absorbance was significantly decreased because of the hydrogen-bond interaction between thiram and TMB, but the addition of Cu^2+^ restored the absorbance intensity. Luminol-H_2_O_2_ chemiluminescence reactions are also used for detecting pesticides in soil. AuNPs or AgNPs can catalyze the decomposition of H_2_O_2_ to produce chemiluminescence in luminol-H_2_O_2_ systems, which can then be combined with aptamers to detect pesticides with specificity (Figure 5b). Li et al. showed, for example, that acetamiprid-induced conformational changes to aptamers lead to the change in morphology (from dispersed to aggregated) of AuNPs. The chemiluminescence signals produced by AuNPs were sensitive enough to detect conformational changes in aptamers prior to and after adding acetamiprid (Figure 5c) [53]. Sun et al. report that AgNPs can not only catalyze the decomposition of H_2_O_2_ but also bond with luminol and aptamers through Ag–S bonds. Atrazine binding specifically to aptamers can significantly weaken ECL intensity by hindering electron transfer between the reaction substrate and electrode. Thus, changes in ECL intensity can be used to detect atrazine concentrations (Figure 5d) [54].

### 2.5. Immobile Substrate

Additionally, nanoparticles can be used as substrates for immobilizing antibodies or enzymes. The gold immunochromatography assay has proven to be the most successful. Using AuNPs-labeled monoclonal antibodies, Zhu et al. developed a one-step strip for detecting triazophos residues. The strips were prepared by dispensing nanocolloidal gold labeled with antitriazophos monoclonal antibody onto a porous glass-fiber conjugate pad. Using ovalbumin hapten as a test line and goat anti-mouse IgG as a control line, nitrocellulose membranes were dispensed with the test and control lines. During binding to colloidal gold-labeled antibodies, triazophos immobilized on the test line will compete with analytes present in samples. Triazophos could prevent colored conjugates from attaching to the test line in sufficient concentrations [55]. The immobilization of molecules is also one function of nanoparticles in electrochemical analysis. Using carboxylated MWCNTs and AchE-labeled Fe_3_O_4_NPs to modify electrodes, Chauhan and Pundir developed a highly sensitive electrochemical biosensor. It has been demonstrated that the biosensor can detect malathion, chlorpyrifos, monocrotophos, and endosulfan linearly [56].

### 2.6. Other Special Functions

The use of nanoparticles with special functions, such as quantum dots’ fluorescence and Fe_3_O_4_ nanoparticles’ magnetic properties, in pesticide detection is also an option. Li et al. prepared an OPs sensor using CdTe QDs with pH-sensitive fluorescence. ACh was hydrolyzed into acetic acid by AChE, which protonated CdTe QDs, decreasing their fluorescence. Because OP inhibits the hydrolysis described above, changes in fluorescence can be inhibited, allowing for the detection of OP [57]. With its ability to be reclaimed through an external magnetic field after the performance, Pan et al. reported magnetic Fe_3_O_4_/SiO_2_-MPS/MIPs via surface molecular imprinting for detecting trace λ-cyhalothrin. Because Fe_3_O_4_/SiO_2_-MPS/MIPs exhibit high fluorescence intensity, low reactivity, and high selective recognition, they were able to detect highly selectively and sensitively λ-cyhalothrin [58].

## 3. Application of Nanoparticle-Based Sensors for Different Pesticides

### 3.1. Insecticides

Insecticides are pesticides that are designed to kill, harm, repel, or mitigate one or more species of insects. A major factor driving agricultural productivity around the world is the use of insecticides, many of which are toxic to humans and/or animals. As the final destination of soil, it is of great significance to monitor the pesticides contained in the soil. Here we are going to introduce nanoparticle-based sensors for organophosphorus, new nicotine, pyrethroids, and organochlorine that are widely used in current agriculture (Table 1).

#### 3.1.1. Organophosphorus Insecticides

Organophosphorus compounds are phosphoric acid esters containing oxygen, nitrogen, carbon, and sulfur [55]. As the most widely used pesticide (40% of pesticide use), organophosphorus has a vital role to play in crop production. Nevertheless, these compounds can be inhaled, ingested, or absorbed through the skin, inhibiting AChE activity and allowing acetylcholine to remain active in synapses. It is known that these compounds can cause central nervous system symptoms, comas, and even respiratory failure in humans. Thus, it is crucial for detecting organophosphorus pesticides.

Methyl parathion, one of the most widely used OPs, was used to control insect pests in a wide variety of crops, including fruits, cereals, nuts, vegetables, and field crops [59]. In light of the widespread and long-term use of methyl parathion, quantitative analyses of its residues have received a lot of attention. In soils, methyl parathion is primarily detected by electrochemical and spectroscopic methods. The excellent electrical conductivity and high surface area of carbon nanoparticles make them ideal for modifying electrodes to reduce LODs. Using a carbon nanoparticle and halloysite nanoclay-modified carbon paste electrode, Srivastava et al. [60] detected methyl parathion by PSA. As a result of the synergistic presence of both modifiers, preconcentration of methyl parathion on the modified electrode surface increased the sensitivity significantly. As a result, the LOD for methyl parathion can reach 0.47 nM and can resist the interference of metal ions and organic molecules. In addition to electrochemical methods, spectroscopic methods such as absorption [18], fluorescence [36], and Raman [61] spectra are also commonly used to detect methyl parathion. Using lanthanum-functionalized AuNPs as a probe, Ai et al. [18] developed a sensitive colorimetric sensor for methyl parathion determination. A change in the absorption spectrum of AuNPs occurs when methyl parathion is combined with lanthanum. In this way, methyl parathion can be detected by a colorimetric assay. There is no need to use complicated instruments to achieve this low LOD (0.1 nM).

Chlorpyrifos is another widely detected OP. Due to its effective insecticidal effects, chlorpyrifos is widely used in agriculture, which results in its widespread residue in the soil. According to studies, chlorpyrifos exposure may affect the brain development of children [62]. A few typical biomolecules, such as AP-algae [63], AChE [56], and HSA [64], serve as special sites for detecting chlorpyrifos. It was found by Jindal et al. [63] that chlorpyrifos inhibits the activity of alkaline phosphatase and weakens the dephosphorylation function of the phosphate monoester by alkaline phosphatase. Therefore, AP-algae-modified ZnO nanoparticles were prepared and used to measure chlorpyrifos by voltammetry and ISFET. In addition to immobilizing AP-algae on the surface, the flower-shaped ZnO nanoparticles enhance electron transfer kinetics and sensitivity. The linear concentration range of this method is from nanomole to molar, and it also has good anti-interference performance. Chlorpyrifos can be detected without external recognition units using Raman spectroscopy, which provides fingerprint information about molecules. Using AuNPs as SERS substrates, Nie et al. [25] determined chlorpyrifos residues in soil. The LOD of the sensor was found to be as low as 10 μg/L. A colorimetric assay using silk fibroin and gold nanocomposite has been developed by Chaudhari et al. [65] Chlorpyrifos can be detected by a color change due to the aggregation of nanoparticles caused by its interaction with gold nanoparticles.

Malathion, a highly volatile organophosphorus pesticide, is widely used in urban and agricultural fields to control insects. Using FRET between Au/Fe_3_O_4_ NPs and rhodamine B, An et al. [39] developed a method for detecting malathion residue in the soil. As a result of the competitive adsorption of malathion and rhodamine B onto Au/Fe_3_O_4_ NPs, malathion concentration was measured with fluorescence at 579 nm. The LOD was as low as 0.59 μM. Furthermore, the Au/Fe_3_O_4_ NPs can be recycled through magnetic concentration washing. The electrochemical aptasensor utilized chitosan-iron oxide nanocomposite film, which was developed by Kaur et al. [66]. As a result of this composite bioelectrode, malathion can be detected by the DPV with a LOD of 0.001 ng/mL.

In addition to the pesticides that have been widely detected, previous reports have also expressed concern about other organophosphorus pesticide residues in soil, including fenthion [67], fenitrothion [68], diazinon [69,70], monocrotophos [56], isocarbophos [17], triazophos [55], methidathion [57], paraoxon-ethy [71], and so on. Various biomolecules are used to recognize pesticides, such as AChE, monoclonal antibodies, and cholinesterase, and detection methods include electrochemistry, fluorescence, and test strips. As a result of these rich recognition units and flexible detection methods, a detection range from nanomoliters per liter to millimoles per liter can be achieved for organophosphorus pesticide residue soil samples.

#### 3.1.2. Neonicotinoid Insecticides

Currently, neonicotinoids represent the most widely used insecticides in the world. In contrast to organophosphorus pesticides, they bind strongly to nAChRs in insects’ central nervous systems. At low concentrations, it stimulates insects’ nervous systems, whereas at high concentrations, it blocks their receptors, paralyzes, or kills them. Insects’ nAChRs are more sensitive to neonicotinoids than vertebrates, hence their selective toxicity. Therefore, they act systemically, traveling through plant tissues and securing all parts of the crop. Nevertheless, broad-spectrum pesticides are not in harmony with long-established integrated pest management principles, which raises concerns about the environment [72].

Since 1994, imidacloprid has been one of the most widely used neonicotinoid insecticides for controlling sucking insects, termites, soil insects, and fleas on pets. In order to monitor soil imidacloprid concentrations, Jaeger et al. [73] developed a SERS-based sensor using AuNPs-modified PLA as the substrate with a LOD of 30 μg/L. In order to further reduce the LOD of imidacloprid, Wang et al. [74] developed an immunoassay using MNPs and UCNPs. MNPs were conjugated with antigens of imidacloprid, while UCNPs were with anti-imidacloprid mAbs. In the competitive immunoassay, antigen-conjugated MNPs were separated by mAb-conjugated UCNPs. The results of the immunoassay showed a LOD of 0.32 µg/L. Due to the high cost and poor stability of monoclonal antibodies, Sharma et al. [75] incorporated MIPs into the imidacloprid sensor. They proposed an electrochemical and fluorescent detection method based on europium-doped superparamagnetic iron oxide nanoparticles. Due to the existence of the nanoparticles that enable the fluorescent dye (fluorescein) to be quenched by imidacloprid, this nanosensor shows a high sensitivity to imidacloprid with a LOD of 10.8 ng/ L. When using an electrochemical method, because the nanoparticle-modified electrode specifically adsorbed imidacloprid in solution, it obtained a 0.0125 μg/L LOD for imidacloprid by the SWSV method.

Acetamiprid is another neonicotinoid insecticide widely used in more than 120 countries. The accumulated residues of acetamiprid in the soil cannot be ignored, even though some countries have prohibited its registration in recent years. By now, AuNPs, MNPs, UCNPs, and some organic NPs have been employed in the detection of acetamiprid in soil. In particular, Belsarec and He et al. [53,76] reported two chemiluminescence-amplified sensing platforms for acetamiprid. As the aptamer has a high affinity for acetamiprid, AuNP or its nanocomposite with GO shows a weakened catalytic effect for chemiluminescence in the presence of H_2_O_2_ and luminol. The LOD of these two nanosensors can reach the picomole level. In order to develop a simple and rapid acetamiprid sensor, Su et al. [37] designed a fluorescent sensing method for acetamiprid. In order to construct the detecting platform, green QDs were deposited onto red QD-doped silica microspheres. As acetamiprid triggers the aggregation of AuNPs, thus inhibiting their fluorescence quenching activity, the concentration of acetamiprid can be determined by the photoluminescence intensity of QDs with a LOD of 16.8 μg/L using IFE. In addition, Wang et al. [41] increased the specificity of the detection of acetamiprid by introducing monoclonal antibodies. In this method, by using AuNPs-coupled antigen as a fluorescence closing reagent and UCNPs-coupled monoclonal antibody as a fluorescent light source, the LOD towards acetamiprid was as low as 0.04 μg/L and it showed no cross-reactivity with other pesticides. Their other work [77] employed aptamers instead of monoclonal antibodies. In detail, aptamers and their complementary DNA were conjugated onto magnet nanoparticles and UCNPs, respectively. Acetamiprid can dissociate UCNPs from MNPs, resulting in a decrease in fluorescence intensity with an external magnet. Consequently, acetamiprid was detected at a LOD of 0.65 μg/L.

Other neonicotinoid insecticides, such as thiacloprid [74] and clothianidin [78], have been studied as well. By using a gold immunochromatographic assay and an enzyme-linked immunosorbent assay, Wang et al. [78] achieved a quantitative/semiquantitative detection of clothianidin in soil with a LOD of 3.8 ng/mL. Another of their works reported a sensitive and rapid immunoassay for the detection of thiacloprid [74]. When using MNPs and UCNPs, thiacloprid LOD can reach 0.61 ng/mL.

#### 3.1.3. Pyrethroids and Organochlorine Insecticides

Pyrethroids are man-made pesticides that mimic the natural pesticide pyrethrum, which comes from chrysanthemum flowers. A pyrethroid’s action is mediated through the interaction with sodium channels and sustained depolarization of neurons. A SERS substrate using AuNPs was prepared by Nie et al. [26] in order to estimate the deltamethrin residue in the soil, a widely used pyrethroid. This method can obtain a LOD of 0.01 mg/L. A fluorescent nanosensor was developed by Wang et al. [79] for the detection of trace alpha-cypermethrin. UCNPs, zeolitic imidazolate frameworks-8, and MIP were self-assembled into a nanocomposite. Alpha-cypermethrin linearly quenched fluorescence intensity with increasing concentrations in the range of 0.10–12 mg/L with a LOD of 0.03 mg/L.

Organochlorine pesticides are a kind of pesticide containing chlorine atoms in their composition. They have been gradually replaced by organophosphorus pesticides and other pesticides because of their danger and persistence. Therefore, current research focuses less on detecting organochlorine residues in soil. Zhang et al. [30] prepared three-dimensional SERS substrates assisted by the self-assembly of magnetic nanoparticles in array pores. In contrast to 2D plasmon structures, 3D SERS substrates have “hot spots” that exist throughout their depth, greatly improving sensitivity. It is possible to detect hexachlorobenzene residues in the soil at levels as low as 10 pM by using the substrate. Dursun et al. [80] succeeded in fabricating an electrochemical sensor by combining nickel nanowires with poly(p-aminophenol)-modified glassy carbon electrodes. This electrochemical sensor can respond to dicofol with a linear range of 0.83–30.7 μM.

**Table 1:** The methods and their performances for the detection of insecticides.

Pesticides	Signals	Used Nanoparticles	Identification Method	Linear Range	LOD	Recovery
Methyl parathion [81]	DPV	Graphene AuNPs	Physisorption	0.95–151.97 μM 1.90–227.95 μM	0.86 μM 2.93 μM	92–113%
Methyl parathion [82]	DPV	MWCNTs-PAAM nanocomposite	Electrocatalysis	0.005–10 μM	2.0 nM	NG
Methyl parathion [83]	DPV	MWCNTs	MIP	0.2–10 μM	67 nM	94.9–106.2%
Methyl parathion [51]	DPV	CuO NPs	Affinity between the Cu and P=S or P=O groups	0.19–5.7 μM	10 nM	80.18–105.48%
Methyl parathion [84]	SWV	AuNPs	Methyl parathion hydrolase	0.075 nM–0.38 μM	0.26 nM	93–107%
Methyl parathion [46]	SWASV	Au–Ag nanoclusters	Electrocatalysis	0.02–8.0 μM 8.0–200 μM	8.2 nM	102.9–104.0%
Methyl parathion [60]	PSA	Carbon NPs and halloysite nanoclay	Electrocatalysis	0.00155–3.67 μM	0.47 nM	NG
Methyl parathion [61]	SERS	Silver/polydopamine/calcium-oxide nanocomposites	SERS	0.01 M–0.9 nM	0.9 nM	NG
Methyl parathion [18]	Colorimetric	AuNPs	Lanthanum	0.5–500 nM	0.1 nM	95.3–107.4%
Methyl parathion [85]	Fluorescence	N-doped CDs	Methyl parathion hydrolase	2.38–73.78 μM	338 nM	95.1–108%
Methyl parathion [29]	SERS	Ag-nanoplate decorated GNS	SERS	1–500 μM	570 nM	NG
Ethyl parathion [60]	PSA	Carbon NPs and halloysite nanoclay	Electrocatalysis	1.21 nM–4.92 μM	0.367 nM	NG
Ethyl parathion [86]	DPV	Carbon nanotube	Intermolecular interactions	0.02–6.50 μM	5.3 nM	97.2–104.6%
Chlorpyrifos [63]	Voltametric and ISFET	Flower shaped ZnO NPs	Alkaline phosphatase	1 nM–0.1 M 0.1 nM–1 mM	1 nM 0.1 nM	96.6–108.9%
Chlorpyrifos [87]	DPAdSV	Ag/Cu alloy NPs	Electrocatalysis	0.01–100 nM	4 pM	85.6–93.4%
Chlorpyrifos [56]	CV	Carboxylated MWCNTs	AChE acetylthiocholine	0.1–50 nM	0.1 nM	NG
Chlorpyrifos [64]	SFI	Pd-doped CdTe QDs MWCNTs	tryptophan residue	0.5 pM–500 nM	0.16 pM	98.5–105.9%
Chlorpyrifos [25]	SERS	AuNPs	SERS	0.01–10 mg/L	10 μg/L	97.5–103.3%
Chlorpyrifos [65]	Colorimetric	AuNPs	Interaction between−P=S group and Au NPs	10–50 ppb	NG	NG
Chlorpyrifos [50]	Absorption spectra	Ag_3_PO_4_ NPs	Oxidase-mimicking	20–80 ppm	9.97 ppm	112.2–164.0%
Chlorpyrifos-methyl [17]	Test paper	Gold NPs	Monoclonal antibodies	NG	0.29 μM	NG
Malathion [66]	DPV	Chitosan-iron oxide nanocomposite	DNA aptamer	0.001–10 ng/mL	1 ng/L	80–92%
Malathion [56]	CV	Iron oxide NPs Carboxylated-MWCNTs	AChE, ATC	0.1–70 nM	0.1 nM	NG
Malathion [39]	FRET	Au/Fe_3_O_4_ NPs	Au−S bond	27.24–99.89 μM	0.59 μM	NG
Fenthion [67]	DPV	Graphene QDs	Pralidoxime	10 pM–0.5 μM	6.8 pM	95.4–104.8%
Fenitrothion [68]	CV	MWCNT	Electrocatalysis	0.01–5.0 mM	6.4 nM	88.0–93.3%
Diazinon [69]	SWASVs	Au–Pt bimetallic nanoclusters	NPs catalyzes	0.01–10.0 μM 10.0–170 μM	2 nM	95.3–105.0%
Monocrotophos [56]	CV	Iron oxide NPs Carboxylated-MWCNTs	AChE ATC	0.1–70 nM	0.1 nM	NG
Triazophos [55]	Test paper	AuNPs	Antibodies	NG	5 ng/mL	NG
Isocarbophos [17]	Test paper	AuNPs	Antibodies	NG	100 μg/L	NG
Methidathion [57]	Fluorescence	CdTe QDs	AChE ATC	0.1–50 ng/mL	0.027 ng/mL	96–105%
Paraoxon-ethyl [71]	Paper strip	Carbonaceous nanoaggregates	Cholinesterase	NG	1.3 ng/mL	90–110%
Imidacloprid [74]	Fluorescence	MNPs UCNPs	Antibody	0.32–299.21 ng/mL	0.32 ng/mL	82.5–102.3%
Imidacloprid [75]	SWSV fluorescence	Iron oxide NPs	MIP	0.059–0.791 μg /L 0.039–0.942 μg/ L	0.0125 μg/ L 0.0108 μg/ L	5.9–100.4%
Imidacloprid [88]	Amperometric current responses	Cu-rGO nanofiber	Electrocatalysis	100–500 nM	2.511 nM	NG
Imidacloprid [89]	CV	MWCNTs	Electrocatalysis	0.2–1.77 μM	0.0374 nM	94–97%
Imidacloprid [73]	SERS	AuNPs	SERS	NG	30 µg/L	NG
Acetamiprid [53]	CL	AuNPs	Aptamer	0.8–630 nM	62 pM	90.4–105.3%
Acetamiprid [21]	Colorimetric	AuNPs	Aptamer	8.7–920 nM	0.56 nM	95.2–104.0%
Acetamiprid [20]	Colorimetric	AuNPs	Aptamer	75 nM–7.5 μM	5 nM	NG
Acetamiprid [77]	Fluorescence	MNPs UCNPs	Aptamer	0.89–114.18 μg/L	0.65 μg/L	78.2–103.5%
Acetamiprid [41]	Fluorescence	UCNPs AuNPs	Antibody	0.002–0.58 µg/L	0.04 µg/L	75.1–104.7%
Acetamiprid [76]	CL	GO/AuNPs	Aptamer	0.221–9 nM	8.9 pM	90.4–108.3%
Acetamiprid [37]	Fluorescence	AuNPs QDs	Cyano group	0.025–5.0 μg/mL	16.8 μg/L	96–105%
Acetamiprid [90]	Chronocoulometry Chronoamperometry	Polypyrrole nanowires	NG	1 ng/L–0.1 g/L 1 pg/L–0.1 ug/L	0.347 pg/mL 0.065 fg/mL	NG
Thiacloprid [74]	Fluorescence	MNPs UCNPs	Antibody	0.61–169.82 ng/mL	0.61 ng/mL	78.4–105.9%
Clothianidin [78]	Test strips	AuNPs	Antibody	3.8–372 ng/mL	3.8 ng/mL 8 ng/mL	78.0–114.5%
Deltamethrin [26]	SERS	AuNPs	SERS	0.01–10 mg/L	0.056 mg/kg	76.0–106.0%,
Alpha-cypermethrin [79]	Fluorescence	UCNPs	MIP	0.10–12 mg/L	0.03 mg/L	83.90–93.15%
Hexachlorobenzene [30]	SERS	Rough ferro-NPs	SERS	NG	~10 pM	NG
Dicofol [80]	DPV	Ni nanowire	Electrocatalysis	0.83–30.7 μM	0.08 μM	95–104.9%
Endosulfan [56]	CV	Iron oxide NPs MWCNTs	AChE	0.1–100 nM	0.1 nM	NG
Carbofuran [91]	DPV	PDDA and GO	Hydrophobic and van der Waals interactions	NG	0.407 μM	101.09 and 96.74%
Carbofuran [26]	SERS	AuNPs	SERS	0.01–10 mg/L	0.01 mg/L	80.0–102.6%,

### 3.2. Herbicides

Herbicides are widely used in the agriculture industry to eradicate weeds and improve crop yields. Despite their beneficial aspects, most herbicides are extremely toxic and nonbiodegradable. Even trace levels of herbicides ingested by humans could result in death and cause health problems. Therefore, herbicide pollution has become a remarkable concern owing to the above-mentioned reasons. Monitoring herbicides accurately, particularly in soil environments, is also crucial to protecting ecological environments [92]. In the following, we primarily introduce several soil residual herbicides, which are currently gaining much attention. These include glycine-derivative herbicides, bipyridines, ditroanilines, triazines, ureas, and others (Table 2).

#### 3.2.1. Glycine Derivative Herbicides

Glycine derivative herbicides are compounds in which the acidic hydrogen ions in glycine are replaced by other groups. Glyphosate is a glycine-derived herbicide that is used extensively in agriculture because it is less harmful to mammals and non-target organisms. In soil and human habitats, glyphosate accumulates due to its solubility and mobility, causing severe environmental pollution and health effects. In recent years, several studies have focused on detecting glyphosate residue in the soil. In these methods, the recognition units are mainly acid phosphatase [93], MIP [94], antibody [95,96], and aptamer [97,98]. Electrochemical spectroscopy, fluorescence, SERS, and electrophoresis can be used to detect glyphosate. For example, a biosensor for the detection of glyphosate based on its inhibition of acid phosphatase was fabricated by Samphao et al. [93]. The biosensor was constructed from carbon electrodes screen-printed with silver nanoparticles, decorated with electrochemically reduced graphene oxide, and immobilized with acid phosphatase. This sensor has a LOD of 0.015 μg/mL. A MIP nanofilm-modified graphite electrode for soil glyphosate detection was prepared by Tiwari et al. [94]. The LOD for glyphosate was as low as 0.35 ng/mL by quantitative differential pulse anodic stripping voltammetry. Additionally, special approaches such as PCR [96], electrophoresis system [99], and resonance Rayleigh scattering [97] can also be employed to detect glyphosate. Zhou et al. [96] synthesized AuNPs probes using anti-glyphosate antibodies and DNA. In order to detect glyphosate without requiring costly and time-consuming experiments, an AuNPs-based biobarcode immuno-PCR was developed. A linear range of 61.1 pg/g to 31.3 ng/g is observed, and a low LOD of 4.5 pg/g is recorded.

#### 3.2.2. Bipyridyliums Herbicides

Bipyridylium herbicides such as diquat and paraquat have been used in approximately 90 countries worldwide due to their superior weed-control abilities. Paraquat is a non-selective weed killer with a very fast uptake rate (50% in the first 10 seconds). Due to its redox activity, paraquat is very dangerous for humans and animals. Even small amounts can lead to Parkinson’s disease, respiratory distress, and neurologic and renal complications. Paraquat has adverse effects on living organisms and the environment, so controlling its level in the soil is crucial. AuNPs have been used in a number of electrochemical detection methods for paraquat due to their effective electrocatalytic performance, and a nanomole-level detection line has been established [100,101,102]. For example, Aliakbar et al. [101] prepared a catex polymeric-AuNPs composite modified electrode for determining paraquat based on the redox property of paraquat. Adding AuNPs to the composite enhanced the electrode’s sensitivity to paraquat measurement. Additionally, AuNPs can be used in SERS for the detection of paraquat with a LOD of 10 nM using a new SERS-active substrate made of Au nanostructures grown on aluminum sheets [103]. Another important direction for the preparation of paraquat nanosensors is the colorimetric method using nanoparticles. Ali and Songsrirote et al. [104,105] realized the detection of micro-molar paraquat by taking advantage of the unique aggregation discoloration effect of AgNPs.

#### 3.2.3. Dinitroanilines Herbicides

Dinitroanilines are a class of compounds that inhibit microtubules, specifically targeting tubulin, a protein found in plants and protists [106]. In spite of its suitability for weeding large-scale crops such as cotton, sugar cane, sunflowers, and soybeans, it is one of the top five most dangerous pesticides [107]. Du et al. [108] synthesized the CDs by employing fresh cherry tomatoes as raw material for the selective detection of trifluralin herbicide in soil samples. Due to trifluralin’s ability to specifically quench CDs’ fluorescence, CDs’ fluorescence intensity was linearly related to trifluralin concentrations in the range of 0.050–200 μM, and the LOD was 0.5 nM. In the same manner, Chowdhury et al. [109] synthesized CDs from polyethylene glycol by adding Ca^2+^ ions. The LOD is 7.89 μM. Menzoori et al. [107] prepared boron nitride quantum dots (BNQDs) to catalyze pyrogallol-H_2_O_2_ reactions. The LOD of the chemiluminescence nanosensor is 6 nM. As well, some nanoparticles with electrocatalytic abilities, such as MWCNTs and copper nanowires [110,111], can greatly enhance the sensitivity of electrochemical detection of trifluralin.

#### 3.2.4. Triazines Herbicides

Triazine pesticides are used in corn, cotton, sorghum, and sugarcane to control weeds both pre-emergence and post-emergence. Both prometryn and atrazine are photosynthesis inhibitors that cause health injuries because they are mutagenic agents as well as endocrine disruptors. Using AgNPs as SERS substrates, Sanchez-Cortes et al. [27] detected prometryn and atrazine. As a result of the strong interaction between the amino groups and oxygenated groups, the sensitivity of SERS was significantly increased, reaching LODs of 1.3 ppb for prometryn and 21 ppt for atrazine. Sun et al. [54] discovered that AgNPs were capable of catalyzing the decomposition of H_2_O_2_ and enhancing ECL intensity. Due to the combination of ATZ and its aptamer, the intensity of the ECL was significantly weakened. The aptasensor had an extremely low LOD of 3.3 × 10^−4^ ng/mL. Additionally, the most abundant metabolite of atrazine herbicide, deethylhydroxyatrazine, was detected by Sanchez-Cortes et al. [28] based on the SERS technique by using AgNPs. The LOD is 34.2 nM. Another triazine herbicide, tribenuron-methyl, was detected by an electrochemiluminescence sensor [112]. An electrode fabricated from a modified glassy carbon and ruthenium complex is highly sensitive and selective due to its regular attachment to silver nanoparticles. The broad linear range from 5.0 pM to 0.6 μM and a low LOD of 1.2 pM were obtained. Furthermore, Luo et al. [113] constructed an electrochemical sensor for the detection of simazine using MIP as the recognition element. In this study, o-aminothiophenol-functionalized AuNPs were used to modify gold electrodes. The electrode demonstrated sensitive and selective detection of simazine herbicide.

#### 3.2.5. Ureas Herbicides

Phenylurea/urea herbicides inhibit photosynthesis in tree cultures, similar to triazine pesticides. Urea herbicides such as diuron are commonly used in crop fields to control weeds. Even so, its high toxicity, including teratogenicity, mutagenesis, carcinogenesis, and genotoxicity, cannot be ignored. Diuron residues in the soil can be detected electrochemically using an NC-modified carbon paste electrode developed by Emmanuel et al. [114]. The linear range of this sensor ranged from 4.2 to 47 μM and the LOD was 0.35 μM.

Another urea pesticide widely used today is linuron, which is effective at eliminating emergent weed seedlings via contact. According to Fatibello-Filho et al. [115], linuron can be determined via DPV utilizing boron-doped diamond electrodes that have been cathodically pretreated. Using optimized DPV conditions, linear analytical curves were obtained for linuron concentrations ranging from 0.61 to 26.0 μM with a LOD of 0.18 μM. Similarly, Kulkarni et al. [116] also developed an electrochemical sensor for the determination of linuron. The carbon paste electrode was fabricated by using MWCNTs along with ZnO NPs. The SWV analysis indicated that the sensor had linear ranges from 0.02 μM to 0.34 μM and a LOD of 5.83 nM.

#### 3.2.6. Diphenyl Ether Herbicides

The extensive use of the diphenyl ether herbicide has caused serious soil pollution problems. As a typical diphenyl ether herbicide, lactofen is urgently required to be detected. Electrochemical voltammetric analysis of the hazardous herbicide aclonifen was performed by Tzu et al. [117]. In this device, glassy carbon electrodes were modified with GdNbO_4_ nanoparticles to obtain electrochemical sensing. By using the DPV method, the LOD was as low as 1.15 nM. Aminabhavi et al. [118] carried out a voltammetric analysis of aclonifen herbicide in soil. A graphitic carbon nitride coating was applied to a glassy carbon electrode to construct the sensing device. Aclonifen at trace levels was detected using the SWV technique.

**Table 2:** The methods and their performances for the detection of herbicides.

Pesticides	Signals	Used Nanoparticles	Identification Method	Linear Range	LOD	Recovery
Glyphosate [93]	CA	AgNPs	Acid phosphatase inhibition	0.05–0.5 μg/mL 0.5–22.0 μg/mL	0.015 μg/mL 2 mg/kg	95.6–104.7%
Glyphosate [94]	DPAnSV	AgNPs	MIP	3.98–176.23 ng/mL	0.35 ng/mL	97.8–102.3%
Glyphosate [99]	Electropherograms	CdTe/CdS QDs	Electrophoretic mobility	77.1–700 mg/kg	25.7 mg/kg	92.0–98.0%
Glyphosate [95]	Fluorescence	CDs; Magnetic NPs	Glyphosate antibody	0.01–80 μg/mL	8 ng/mL	87.4–103.7%
Glyphosate [119]	Fluorescence	CdTe QDs; Au NPs	Electrostatic interactions,	0.02–2.0 μg/kg	9.8 ng/kg	88.5–102.6%
Glyphosate [98]	SERS	Au NPs	SERS	0.003–0.07 nM	0.002 nM	92.3–105.3%
Glyphosate [120]	SERS	rGO, AgNPs, TiO_2_ nanotube	SERS	10^−2^–10^−12^ M	3 µg/L	100.2–103.1%
Glyphosate [96]	Bio-barcode immuno-PCR	AuNPs	Glyphosate antibody	61.1 pg/g–31.3 ng/g	4.5 pg/g	99.8–103.7%
Glyphosate [121]	CL	ZnO NPs	[Ru(bpy)_3_]^2+^	1–10 μM	300 nM	92%
Glyphosate [97]	Resonance Rayleigh scattering	Gold-doped polystyrene nanoenzyme	Aptamer	0.5–20 nM	0.24 nM	NG
Paraquat [101]	Ad-DPCSV	AgNPs	Redox activity of paraquat	19–1000 nM	0.23 nM	99–102%
Paraquat [100]	SQW	AuNP-MWCNT	Electrocatalysis	1.0–2.0 μM	32 nM	93.5–101.6%
Paraquat [102]	DPV	AuNPs	Electrostatic interactions	7.0–1500 nM	0.2 nM	95.0%
Paraquat [103]	SERS	AuNPs	SERS	NG	10 nM	NG
Paraquat [104]	Colorimetric	AgNPs	Forming charge transfer complexes	20–180 µM	6.27 µM	NG
Paraquat [105]	Colorimetric	AgNPs	Coulombic attraction	0. 194–194 µM	0.05 mg/L	89.5% and 86.6%
Trifluralin [109]	Fluorescence	CDs	Fluorescence Quenching	NG	7.89 μM	NG
Trifluralin [108]	Fluorescence	CDs	Fluorescence Quenching	0.050–200 μM	0.5 nM	94.6–103.2%
Trifluralin [107]	CL	BNQDs	Nanocatalysts	0.02–90 µM	6.0 nM	94–104%
Trifluralin [122]	CV	MWNTs	Electrocatalysis	5–6000 nM	2.0 nM	96.7–101.0%
Trifluralin [110]	FFT-SWV	Copper nanowire	Electrocatalysis	100–0.02 nM	0.008 nM	99.3–101.5%
Trifluralin [111]	SWV	MWCNT Fe_3_O_4_/SiO_2_ NPs	Electrocatalysis	0.01–8 μM	3 nM	NG
DEHA [28]	SERS	AgNPs	SERS	2.04–163 μM	34.2 nM	NG
Prometryn [27]	SERS	AgNPs	SERS	NG	5.6 nM	NG
Atrazine [27]	SERS	AgNPs	SERS	NG	0.1 nM	NG
Atrazine [54]	ECL	AgNPs	Aptamer	1 pg/mL–10 μg/mL	0.33 pg/mL	89.13–123.03%
Tribenuron-methyl [112]	ECL	AgNPs BNQDs	Cooperation effect	5.0 pM–0.60 μM	1.2 pM	98.2–100.8%
Simazine [113]	CV	AuNPs	MIP	NG	0.013 μM	91.4–96.8%
Diuron [114]	SWV	NC	Electrocatalysis	4.2–47 µM	0.35 µM	96%
Linuron [115]	DPV	PtNPs	Electrocatalysis	0.61–26.0 μM	0.18 μM	90.9–104%
Linuron [116]	SWV	MWCNTs/ ZnO NPs	Electrocatalysis	0.02–0.34 μM	5.83 nM	96.2–99.42%
Aclonifen [117]	DPV	GdNbO_4_ NPs	Electrocatalytic	0.02–78 μM	1.15 nM	80–92.5%
Aclonifen [118]	SWV	g−C_3_N_4_	Electrocatalytic	0.01–1.2 μM	1.28 nM	97.4–98.7%

### 3.3. Fungicide

Pesticides that kill parasitic fungi and their spores are called fungicides. Because fungi can severely damage agriculture, causing losses in yield, quality, and profits, fungicides are widely used in agriculture to combat fungal infections. Fungicides, however, pose a risk to aquatic biota and are highly toxic to a broad range of organisms [123]. For this reason, fungicide detection has been the focus of research over the past decade. As part of our review, we discussed nanoparticle-based sensors for detecting carbamates, triazoles, and other kinds of fungicides in soil (Table 3).

#### 3.3.1. Carbamates

Carbamate fungicides are derivatives of carbamic acid and have carbamate ester bonds as functional groups. These compounds act reversibly by inhibiting AChE activity in the nervous system. Thiram is one of the most widely used carbamate fungicides for preventing various crop diseases. However, its overuse causes soil pollution, seeps into groundwater, mixes with air dust, and can even have highly toxic effects on human skin and mucosa. Numerous pieces of research have focused on developing simple and sensitive nanosensors to detect residues of thiram in soil using SERS, absorption spectra, and colorimetric electrochemical spectra. Disulfide bonds have a strong affinity for noble metal NPs; therefore, many experiments have used noble metal NPs as SERS substrates to enhance Raman spectral signals [31,32,33,35,124]. For example, Wang et al. [34] prepared dense gold nanospikes on magnetic microparticles by using AgNPs-modified magnetic microparticles as a template. Due to the abundance of hotspots, the prepared substrate shows excellent SERS sensitivity for the detection of thiram in soil with a LOD of 1 nM. In our previous study [13], we proposed a highly sensitive and selective SERS substrate based on TSNPs with small sizes and sharp corners. The method exhibits a linear response from 0.12 to 4.8 μg/g with a low LOD of 90 ng/g. Another universal method to detect thiram in soil is through colorimetric sensors. By synthesizing AuNP encoded with 4-aminothiophenol, Liang et al. [22] developed a colorimetric sensor. The silver ions that trigger AuNP aggregation were inhibited by the competitive reaction between thiram and silver ions. In a linear range of 0.05–2.0 μM, the thiram concentration can be detected by monitoring the color change of the probe. In our previous work [23], we developed an anti-etching colorimetric detection method based on TSNPs. As a result of regulating the antagonistic relationship between thiram protection and etching, the LOD of this method is 19.7 nM for the linear range of 0.025–0.35 μM.

Ziram is another dithiocarbamate fungicide used to treat a broad range of fungi and diseases in plants. Rao et al. [52] synthesized Pt/Co_3_O_4_ NPs by precipitation and reduction methods to analyze ziram residues in soil. Due to the superior oxidase activity of Pt/Co_3_O_4_ NPs, thiram and ziram have been detected successfully with LODs of 0.065 μM and 3.36 μM. Pourreza et al. [125] also developed an absorption spectroscopic approach for detecting ziram with dispersive liquid-liquid microextraction. As ziram can affect the formation of gold nanoparticles, a relationship was established between the absorbance change of the reaction solution and ziram. Detailed colorimetric data for ziram can be obtained in the concentration range of 0.12–2.52 ng/mL with a LOD of 0.06 ng/mL.

Carbendazim is also a widely used broad-spectrum carbamate fungicide. A large number of studies have found that the electrochemical method based on nanoparticle enhancement has good performance in the detection of carbendazim [47,126,127,128,129]. Aminabhavi et al. [128] realized the detection of carbendazim at the trace level by employing GO and graphitic carbon nitride nanohybrid electrode assemblies. By using SWV techniques, the LOD of carbendazim can reach 2.82 nM. Srivastava et al. [129] fabricated a GNS and amberlite XAD-2 modified electrode for voltammetric determination of carbendazim. The concentration of carbendazim can be determined in the range of 8.36 × 10^−9^ to 4.13 × 10^−6^ M.

#### 3.3.2. Triazole Fungicides

A triazole fungicide, tebuconazole, treats pathogenic fungi in agriculture. As a carcinogen, it shows a potential risk to the environment even at very low concentrations. Toi et al. [130] describe a simple platform using an aptamer as the bioreceptor. As the pesticide interacts with the aptamers on the nanoparticles’ surface, they can detach from the nanoparticles. When high salt concentrations are present, nanoparticles aggregate, and the absorption spectrum changes. Unaided eye monitoring of tebuconazole in the soil is possible at a LOD of ~10 nM.

Using mesoporous, structured MIP sensors, Jalili et al. [131] detected diniconazole, a triazole fungicide that inhibits the demethylation of steroids and disrupts ergosterol biosynthesis. In this probe, a carbon-doped silica core is used as a reference and compensates for environmental effects. Mesoporous silica contains CdTe/CdS QDs, which provide an analytical signal. CdTe/CdS QDs are selectively quenched by diniconazole, resulting in a color change from green to blue. Sensor response ranges from 20 to 160 μg/L with a LOD of 6.4 μg/L.

#### 3.3.3. Others

Chroneb is an endogenic fungicide with special effects on cotton, tobacco, pepper, tomato, and other crops with blight. Li et al. [132] synthesized a composite of ZnS rods and CoS NPs modified with MIPs. Chloroneb could be detected rapidly, sensitively, and highly selectively with the DPV. In optimal conditions, the concentration of chloroneb can be measured using the oxidation peak current in the concentration range of 0.003 to 0.2 μM and 0.2 to 3.2 μM with a LOD of 0.87 nM.

Cymoxanil, an aliphatic nitrogen fungicide widely used on vegetables and fruits, can cause sudden and acute poisoning in humans. Cymoxanil can be visualized colorimetrically and fluorometrically using the IFE between AgNPs and CDs, as described in Gui et al. [38]. Due to its electrostatic attraction and hydrogen bonding effects, cymoxanil triggers AgNP aggregation when added to the mixture. An overlapping spectrum is observed between AgNPs and CDs after aggregation. As for the LODs, colorimetry can reach 3 nM while fluorescence can reach 2 nM.

Chlorothalonil, a substituted benzene fungicide, has become the second-most widely used fungicide. In nature, chlorothalonil and its metabolites have both water solubility and difficult degradability. Based on the IFE between AuNPs and ratiometric fluorescent QDs, Dai et al. [133] prepared a sensitive sensor for detecting chlorothalonil residues in soil. The addition of chlorothalonil can inhibit the activity of papain, which hydrolyzes protamine to restore the fluorescent signal by quenching fluorescence. Based on this sensing platform, chlorothalonil is detected at levels as low as 0.0017 ng/mL and ranges from 0.34 to 2320 ng/mL.

Thiabendazole is widely used to prevent and treat sclerotium blight, downy mildew, and root rot in plants. Because chlorothalonil is used indiscriminately, excess residues are present in soil from chlorothalonil use; therefore, chlorothalonil residues should be monitored. Qu et al. [134] prepared an AuNPs-based SERS substrate to detect chlorothalonil residue in the soil. It was found that Raman peak intensity and chlorothalonil concentration in soil were linearly correlated, and the LOD was 8.1 nM.

**Table 3:** The methods and their performances for the detection of fungicide.

Pesticides	Signals	Used Nanoparticles	Identification Method	Linear Range	LOD	Recovery
Thiram [33]	SERS	Cu_2_O nano-octahedrons	SERS	10^−3^–10^−7^ M	0.48 ng/g	NG
Thiram [31]	SERS	Rough Au NRs	SERS	0.0192–0.96 µg/g	0.0005 ppm	NG
Thiram [124]	SERS	Au@Ag nanocube	SERS	0.24–4.8 mg/kg	0.148 mg/kg	NG
Thiram [32]	SERS	PDA@Au nanowaxberry	SERS	NG	0.31 μg/g	NG
Thiram [34]	SERS	Au nanospikes on magnetic microparticles	SERS	10^−5^–10^−8^ M	10 pM	NG
Thiram [35]	SERS	AuNPs	SERS	0.1–12 μg/g	50 ng/g	91.76–112.3%
Thiram [13]	SERS	TSNP	SERS	0.12–4.8 μg/g	90 ng/g	93–111.75%
Thiram [22]	Colorimetric	AuNPs	Competitive reaction between thiram and Ag^+^	0.05–2.0 µM	0.04 μM	80–90%
Thiram [23]	Colorimetric	TSNPs	Ag–S bonds	0.025–0.35 μM	19.7 nM	94.7–97.5%
Thiram [52]	Absorption spectra	Pt/Co_3_O_4_ nanoflowers	Oxidase-like activity	0.6–250 µM	0.065 µM	95.33–101.60%
Ziram [125]	Absorption spectra	AuNPs	Ziram influenced the formation of AuNPs	0.12–2.52 ng/mL	0.06 ng/mL	95.1–103.9%
Carbendazim [127]	CV	AuNPs	Electrocatalytic	0.05–25 μM	2.9 nM	100.1–103.3%
Carbendazim [47]	Amperometric response	Pd NPs	Electrocatalytic	0.02–35 μM	3 nM	99.7–108.1%
Carbendazim [129]	Adsorptive Stripping DPV	GNs	Electrocatalytic	8.36 nM–4.13 μM	3.14 nM	98.33–99.70%
Carbendazim [126]	CVs	Carbon nanofiber Cu NPs	Electrocatalytic	0.8–277.0 µM	28 nM	97–99.5%
Carbendazim [128]	SWV	GO/g-C_3_N_4_ nanohybrids	Electrocatalytic	1.0 × 10^−8^–2.5 × 10^−4^ M	2.82 nM	97.85–98.2%
Cymoxanil [38]	Ratiometric colorimetry	AgNPs	Electrostatic attraction hydrogen bonding	0.01–0.8 μΜ	3 nM	NG
Cymoxanil [38]	Ratiometric FL	AgNPs	Electrostatic attraction hydrogen bonding	0–0.15 μg/mL	2 nM	97–105%
Chlorothalonil [133]	Ratiometric fluorescent	AuNPs CdTe QDs	Electrostatic attraction	0.34–2320 ng/mL	0.34–2320 ng/mL	91.8–104.4%
Tebuconazole [130]	Colorimetric assay	AgNPs	Aptamers	25–250 nM	10 nM	89.90–110.86%
Diniconazole [131]	Fluorescence	CdTe/CdS QDs	MIP	20–160 µg/L	6.4 µg/L	95.6–105.5%
Chloroneb [132]	DPV	CoS NPs attached ZnS rods	MIP	0.003–0.2 μM 0.2–3.2 μM	0.87 nM	95.7–101.2%
Chlorantraniliprole [134]	DPV	Carbon nanotube with thiophene-ferrocene moieties.	NG	0.01–7.00 μM	8.1 nM	102.4–104.8%

## 4. Conclusions and Outlook

In the past 10 years, sensors based on nanomaterials for soil pesticide residue have been greatly developed. In these systems, nanoparticles can play two main roles: one is signal conversion, and the other is signal enhancement. In the signal conversion, due to the interaction between the incident illumination and the nanoparticles produced by the LSPR, the nanoparticles process a unique color, and the color is determined by the shape and aggregation state of the nanoparticles, so the concentration of pesticides can be indicated by the color of the nanoparticles. Other special nanoparticles, such as quantum dots or upconverted nanoparticles, can also be used to indicate the content of pesticides by photoluminescence. In the aspect of signal enhancement, the LSPR phenomenon of the nanoparticle can not only enhance or quench the fluorescence but also enhance the Raman signal of pesticide molecules. The high specific surface area and conductivity of nanoparticles can greatly improve the detection sensitivity by acting as nanoenzymes and electrocatalysts. By conjugating some identification units such as an antibody, aptamer, or MIP to these nanoparticles, a variety of nanoparticle-based sensor platforms was developed, and they have been widely used in soil pesticide detection of insecticides, herbicides, fungicides, and other pesticides.

Despite the great advances that have been achieved in nanoparticle-based sensors for soil pesticide detection, current studies still have several limitations that should be further addressed. Firstly, at present, the nanosensor for the detection of insecticides is very popular, but the detection of herbicides and fungicides, which are also various and harmful, is relatively small. Compared with fungicides and herbicides, insecticides have a larger amount of usage and were commercialized earlier, thus receiving more attention. Moreover, pests pose a greater threat to crops than weeds and fungi, leading to greater public awareness of the use of insecticides. Therefore, the detection of insecticides is more likely to attract people’s attention. Additionally, the metabolites of some pesticides may be toxic [135], but their detection is rarely reported. Second, most methods require load operation steps, complex operation methods, and expensive instruments or equipment, and some methods require stringent storage conditions, which are not friendly to ordinary users. Therefore, it is of great significance to further develop miniaturized, intelligent, and simplified pesticide detection methods such as colorimetry and test paper methods. Finally, soil, as the destination of pesticides, contains a wide variety of pesticides, but most detection methods can only detect one or two pesticides at the same time. Therefore, in the future, it may be necessary to combine techniques using nanomaterials with high-throughput chips to realize the simultaneous detection of multiple pesticides.

## Data Availability

No new data were created.

## Abbreviations

Ach	Acetycholine
AChE	Acetylcholinesterase
AgNPs	Silver/Ag nanoparticles
AP-algae	Alkaline phosphatase
ATC	Acetylthiocholine
AuNRs	Gold/Au nanorods
AuNPs	Gold/Au nanoparticles
BNQDs	Boron nitride quantum dots
BSA	Bovine Serum Albumin
CA	Chronoamperometry
CDs	Carbon dots
CL	Chemiluminescence
DEHA	Deethylhydroxyatrazine
CNPs	Carbon nanoparticles
DPAdSV	Differential pulse adsorptive stripping voltammetric
DPAnSV	Differential Pulse Anodic Stripping Voltammetry
DPV	Differential pulse voltammetry
ECL	Electrochemiluminescence
Fe_3_O_4_NPs	iron oxide nanoparticles
FRET	Fluorescence resonance energy transfer
g-C_3_N_4_	Graphitic carbon nitride
GdNbO_4_	Gadolinium niobate
GNS	Graphene nanosheets
GO	Graphene oxide
HAS	Human serum albumin
HNC	Halloysite nanoclay
IFE	Inner filter effect
IPM	Integrated pest management
ISFET	Ion sensitive field effect transistor
LOD	Limit of detection
LSPR	Localized surface plasmon resonance
mAb	Monoclonal antibody
MIPs	Molecularly imprinted polymers
MNPs	Magnetic nanoparticles
MPS	3-(methacryloxyl) propyl trimethoxysilane
MWCNTs	Multiwalled carbon nanotubes
nAChRs	Nicotinic acetylcholine receptors
NC	Nanocrystalline cellulose
NPs	Nanoparticles
NVs	Nanovines
Ops	Organophosphorus pesticides
PAAM	Poly(acrylamide)
Pd NPs	Palladium nanoparticles
PDA	Polydopamine
PLA	Polylactic acid
PRM	Prometryn
PSA	Potentiometric stripping analysis
QDs	Quantum dots
RF-QDs	Ratiometric fluorescent quantum dots
RhB	Rhodamine B
SEF	Surface enhanced fluorescence
SERS	Surface enhanced Raman spectroscopy
SFI	Single frequency impedance
ssDNA	Single-strand DNA
SWASV	Square wave anodic stripping voltammetry
SWSV	Square wave stripping voltammetry
SWV	Square wave voltammetry
TMB	3,3,5,5-Tetramethylcyclohexanone
TSNPs	Triangular silver nanoplates
UCNPs	Upconversion nanoparticles
